# Genome-Wide Analysis of Copy Number Variation in Type 1 Diabetes

**DOI:** 10.1371/journal.pone.0015393

**Published:** 2010-11-15

**Authors:** Britney L. Grayson, Mary Ellen Smith, James W. Thomas, Lily Wang, Phil Dexheimer, Joy Jeffrey, Pamela R. Fain, Priyaanka Nanduri, George S. Eisenbarth, Thomas M. Aune

**Affiliations:** 1 Department of Microbiology and Immunology, School of Medicine, Vanderbilt University, Nashville, Tennessee, United States of America; 2 Department of Medicine, Division of Rheumatology, School of Medicine, Vanderbilt University, Nashville, Tennessee, United States of America; 3 Department of Biostatistics, School of Medicine, Vanderbilt University, Nashville, Tennessee, United States of America; 4 Functional Genomics Shared Resource, School of Medicine, Vanderbilt University, Nashville, Tennessee, United States of America; 5 Barbara Davis Center for Childhood Diabetes, University of Colorado, Aurora, Colorado, United States of America; La Jolla Institute of Allergy and Immunology, United States of America

## Abstract

Type 1 diabetes (T1D) tends to cluster in families, suggesting there may be a genetic component predisposing to disease. However, a recent large-scale genome-wide association study concluded that identified genetic factors, single nucleotide polymorphisms, do not account for overall familiality. Another class of genetic variation is the amplification or deletion of >1 kilobase segments of the genome, also termed copy number variations (CNVs). We performed genome-wide CNV analysis on a cohort of 20 unrelated adults with T1D and a control (Ctrl) cohort of 20 subjects using the Affymetrix SNP Array 6.0 in combination with the Birdsuite copy number calling software. We identified 39 CNVs as enriched or depleted in T1D versus Ctrl. Additionally, we performed CNV analysis in a group of 10 monozygotic twin pairs discordant for T1D. Eleven of these 39 CNVs were also respectively enriched or depleted in the Twin cohort, suggesting that these variants may be involved in the development of islet autoimmunity, as the presently unaffected twin is at high risk for developing islet autoimmunity and T1D in his or her lifetime. These CNVs include a deletion on chromosome 6p21, near an HLA-DQ allele. CNVs were found that were both enriched or depleted in patients with or at high risk for developing T1D. These regions may represent genetic variants contributing to development of islet autoimmunity in T1D.

## Introduction

Type 1 diabetes (T1D) results from immune-mediated selective destruction of pancreatic islet cells resulting in insulin deficiency and hyperglycemia [1], [2]. Symptoms of polydipsia, polyuria, polyphagia and weight loss manifest when significant numbers of islet cells have been destroyed. However, antibodies to islet autoantigens can be detected in peripheral blood prior to clinical disease[1], [3]. With early diagnosis of disease or assessment of risk, immune therapy may impede islet destruction and preserve insulin production, delaying onset of clinical manifestations[2].

Another component of T1D that aids in our understanding of the disease and assessment of risk is genetic inheritance. A long-term (up to 40 year) study of twin pairs in Finland revealed a monozygotic (MZ) pairwise concordance for T1D of 27.3% while the concordance for dizygotic (DZ) twins was 3.8%[4]. The impact of genetics was further made clear in this study because upon diagnosis of T1D in one twin, the length of time to diagnosis in the other twin in the concordant pairs was a maximum of 6.9 years in MZ twins and 23.6 years in DZ twins[4]. In addition to measuring incidence of T1D in twin studies, islet antigen-specific autoimmunity can also be determined. As a precursor to T1D, autoimmunity is defined as the presence of antibodies to islet autoantigens in sera[5]. In another study, 83 unaffected monozygotic twins were followed for nearly 44 years and incidence of autoimmunity or diagnosis of T1D was recorded. This study showed a 65% cumulative incidence of T1D by 60 years of age and more than 75% tested positive for an islet autoantibody during the course of the study. Once autoimmunity was established, the risk of diabetes was 89% within 16 years of the first positive autoantibody test.

Clearly genetics play an important role in the T1D disease process as both MZ and DZ twins have the same environmental exposures but different concordance rates and length to diagnosis of the second twin. Numerous genes have been associated with T1D, the most significant being the HLA region on chromosome 6[6]. More than 90% of type 1 diabetics carry HLA alleles DR3-DQ2 or DR4-DQ8 compared to no more than 40% of the general population[7]. Alleles at HLA-DQB1 are known to be, in part, protective[8]. Single nucleotide polymorphisms (SNPs) are also associated with T1D. A recent genome-wide association study of approximately 2,000 patients with each of 7 common, chronic diseases, including T1D, and 7,000 shared controls confirmed the association of SNPs in 5 previously identified regions with T1D and discovered 5 novel associations. However, the authors concluded that these regions, with the exception of the HLA on chromosome 6, confer only modest effects on T1D, and “the association signals so far identified account for only a small proportion of overall familiality”[9]. These results suggest that additional genetic variants contribute to inheritance of T1D.

Another class of genetic variation is the amplification or deletion of >1 kilobase segments of the genome, also called copy number variations (CNVs)[10], [11]. Gene duplications were first identified in the pathogenesis of Charcot-Marie Tooth disease in the 1980s; a copy number amplification of the PMP22 gene was shown to be sufficient to cause disease[12]. These regions of variance were thought to be rare and when the human genome was published, variance amongst humans was primarily attributed to base-pair level SNPs[13], [14]. However, copy number variants were discovered to be present and widespread in the genome shortly thereafter[10], [11]. These variants are generated during normal recombination events, leading to inherited CNVs, as well as somatically throughout life in rapidly dividing cells[15], [16], [17]. CNVs can directly influence gene expression through dosage effects where more copies of the gene produces greater expression, and also by altering the transcriptional regulation of the genome, both of the region of variance itself and regions up to 1 megabase away[18], [19], [20].

Monozygotic twins discordant for disease represent a controlled population in which to identify potentially disease-associated CNVs. Monozygotic twins do not have identical genetic sequences and are known to vary in CNVs and at the epigenetic level[21], [22], [23], [24]. Differences may arise during prenatal cell division or post-natally in continuously dividing cells like lymphocytes. The latter would result in CNVs that not only differ from the co-twin but also from CNVs in other cells and tissues of the body. In the case of disease discordant monozygotic twins, if a CNV were associated with a certain disease, we presume the twin affected by the disease would have the variant and the unaffected twin would not. A study of nine MZ twin pairs discordant for Parkinson's disease identified 35 regions of variance present in only the affected twin of at least four of those pairs, confirming the hypothesis that MZ twins differ in CNVs and that these regions may be involved in the development of disease, as evidenced by the presence of specific CNVs in multiple affected twins[22].

There are numerous other diseases and states associated with differences in CNVs, among them schizophrenia and bipolar disorder[25]. But not all CNV associations are with neurologic or behavioral diseases. Recent studies have shown additional functional implications of CNV and disease, notably in studies of CNV of the Fc-gamma receptor and the autoimmune disease systemic lupus erythematosus (SLE). Patients with SLE are more likely to have fewer copies of *FCGR3B*, encoding a protein involved in the uptake and clearance of immune complexes[26].

We hypothesize that CNVs contribute to susceptibility to and/or protection from T1D. To test this hypothesis, we performed genome-wide analysis on a cohort of 20 unrelated adults diagnosed with T1D and 20 unrelated control (Ctrl) subjects to identify CNVs either enriched or depleted in the T1D cohort compared to Ctrl. We then looked at the frequency of these variants in a second cohort of 10 MZ twin pairs disease discordant for T1D. The frequencies of the CNVs of interest did not differ from the affected twin subset to the unaffected twin subset. However, because of the high lifetime incidences of autoimmunity and/or T1D in the unaffected twins, the 10 twin pairs were considered as a single cohort with or at high risk for T1D[3]. This analysis identified 5 CNVs enriched and 4 CNVs depleted in both the T1D and Twin cohorts.

## Results

We sought to determine if CNVs are associated with T1D by performing genome-wide CNV analysis on a cohort of 20 patients with T1D and 20 Ctrl patients using the Affymetrix SNP Array 6.0. An additional cohort of 10 monozygotic twin pairs discordant for T1D was analyzed for validation purposes. Quality of the hybridization, as defined by Affymetrix in the Genotyping Console as a contrast QC <0.4, was assessed and 1 Ctrl sample failed prior to copy number analysis.

**Figure 1:**
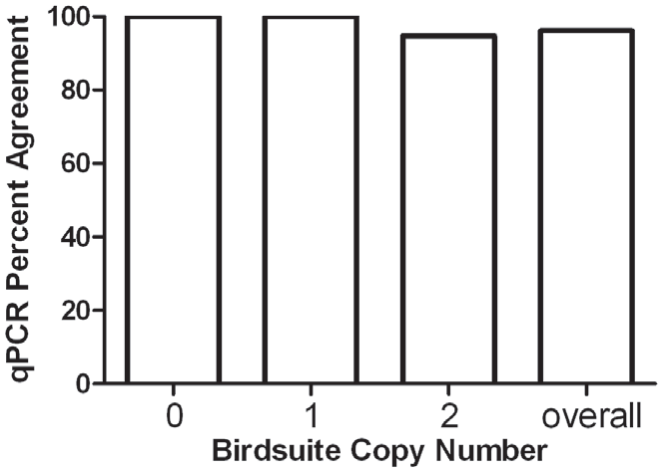
Percent agreement between Birdsuite copy number calls and qPCR. Percent agreement between the Affymetrix array copy numbers, as determined by the Birdsuite software, and qPCR copy number, determined using ΔΔCt calculations, is plotted for each copy number class. Percents are based on 214 comparisons from copy numbers for 37 samples on 6 distinct chromosomal regions (7q33, 8q11, 8q24, 15p13, 16p12). For copy number 0, 6/6 samples agreed (100%). For copy number 1, 45/45 samples agreed (100%). For copy number 2, 127/134 of samples agreed (94.7%). The overall agreement is 178/185 samples (96.2%).

Of primary importance in the analysis of these data was the validity of our copy number calling algorithm. Raw data from all 3 cohorts, 59 Affymetrix arrays in total, were inputted into the Birdsuite programs and copy numbers were called across the genome. The Birdsuite software determined integer copy numbers of predefined regions of common variance (copy number polymorphisms, CNPs) and employed a more complex, multi-dimensional model to identify rare variants [27]. Output files contain copy number values across the chromosome with a confidence score of each individual call (Table S1). Genome wide call rates were also estimated for each individual sample. Two samples from the unrelated adult T1D cohort failed a quality control checkpoint of call rate greater than 98%. The remaining 57 arrays had call rates ≥ 98.6%. We compared copy numbers determined by the Birdsuite analysis to copy numbers determined by quantitative PCR (qPCR) in 37 samples of genomic DNA across 5 distinct chromosomal regions (Table S2). For qPCR experiments, Applied Biosystem's CopyCaller1.0 program determined a non-integer copy number based upon the ΔΔCt calculation and then predicted an integer copy number, each with an associated confidence value. For 185 separate experimental points, there was >96% agreement in copy number determinations made by the Birdsuite analysis and the qPCR analysis (Figure 1).

For the analysis, we first catalogued all confident, variant CN calls on chromosomes 1–22 within the framework of known copy number polymorphisms (CNPs) [28]. CNPs are regions of copy number variance present in greater than 1% of the 270 HapMap samples, resulting in a library of 1,320 CNPs. Novel CNVs not represented in the CNP library were also identified and included in the analysis.

A single CNV is capable of causing disease. In the case of Charcot Marie Tooth disease type 1, 70% of patients have one singular pathogenic variant[12], [29]. In a more common disease, like T1D, we hypothesized that a single undiscovered variant would not be present and pathogenic at a percentage as high as 70%, rather we set the threshold of variance within each diabetes group at roughly half that, or 40%. Additionally, to ensure selection of variants differentially expressed between the two groups, we further limited the classification of enrichment in T1D to those CNVs present at a 1.5 fold greater frequency than Ctrl. Conversely, a CNV was classified as depleted in T1D if it was present in >40% of the Ctrl cohort at a 1.5 fold greater frequency than T1D.

Variants in the T1D group were compared to those in the Ctrl group and 18 CNPs present in > 40% of the T1D cohort at a 1.5 fold greater frequency than the Ctrl cohort were identified as enriched in T1D. Conversely, 20 CNPs and 1 novel CNV were depleted in the T1D cohort, defined as a variant present in >40% of the Ctrl cohort at a 1.5 fold greater frequency than the T1D cohort. These 39 CNVs were then studied in a second cohort.

The Affymetrix chip determines copy number based on values of nearly 1,000,000 probes in the genome, resulting in a high probability for both type I and II errors. To help control for these errors, we performed genome-wide copy number analysis in a second cohort of patients, monozygotic twin pairs discordant for T1D. We hypothesized that the 39 CNVs identified in the first T1D:Ctrl comparison may be differentially present in this second cohort of MZ twins. For each twin, confident variant calls were catalogued in the CNP library as before. CNVs present in only 1 twin of a pair were isolated and grouped based on disease status (affected or unaffected). These variants were compared to the 39 CNVs from the previous analysis and no overlap was found. Additionally, there were no CNVs present in more than 2 affected or unaffected twins of the pairs when this cohort was considered independently of the previous analysis.

The unaffected twin in each of these MZ twin pairs will have a greater than 75% lifetime incidence of developing islet autoantibodies and 65% of these now-unaffected twins will go on to develop T1D in their lifetime[3]. As such, CNVs may be enriched in this group as a whole that confer risk to developing islet autoimmunity or T1D. Alternatively, the CNVs depleted in the unrelated adult T1D cohort may also be depleted in the twin cohort as a whole. The 10 MZ twin pairs were compared to the Ctrl cohort to determine CNVs that were enriched or depleted in the Twin group. Criteria for enrichment and depletion were identical to those outlined above. A total of 49 CNPs were enriched and 23 CNVs were depleted in the Twin cohort. Of the depleted CNVs, 22 were CNPs while 1 novel CNV was identified. All together, 72 CNVs were enriched or depleted in the Twin cohort.

The 72 CNVs identified in the Twin cohort were compared to the 39 CNVs identified in the adult T1D cohort to identify CNVs present in both cohorts. Of these, 10 CNVs were enriched in both cohorts relative to Ctrl and 11 CNVs were depleted. Based upon permutation testing (with 1000 permutations), the p-value or probability of observing 10 or more overlapping CNVs in these cohorts by chance is 0.005 ( = 5/1000).

The 21 CNVs were further classified to select those CNVs greater than 1,000 base pairs in length and identified by at least 3 consecutive probes on the Affymetrix array. A total of 9 CNVs (8 CNPs, 1 novel CNVR) met these criteria. Of these, 5 CNPs were enriched in the T1D and Twin cohorts and are identified by their CNP ID (Table 1). These CNPs are located on 5 different chromosomes, range in size from 1,400 base pairs to 14,000 base pairs and are deletions to copy numbers of 0 or 1. Frequencies in the T1D and Twin cohorts range from 50% to 95% with corresponding frequencies in the Ctrl cohort from 21% to 58%. CNP253, on chromosome 2p11, contains part of a non coding RNA, NCRNA00152. CNP1303 contains the gene *SNTG1*, encoding gamma syntrophin, a cytoplasmic peripheral membrane protein known to be expressed in brain. Two regions, CNP934 and CNP1162, contain at least one segment of DNA longer than 100 bp with more than 70% evolutionarily conserved sequence to *mus musculus* as determined by the ECR browser, defined as an evolutionarily conserved noncoding sequence[30]. Each of these sequences encodes at least one potential transcription factor binding site suggesting these regions may have regulatory function. The sequence encompassed by CNP1956 is not gene coding and does not contain an evolutionarily conserved noncoding sequence.

**Table 1:** CNVs enriched in T1D and Twin cohorts, relative to Ctrl.

CNP ID^a^	Chr	Start	End	Amplification or Deletion	Ctrl%	T1D%	Ctrl:T1D *p* b	Twin%	Ctrl:Twin *p*	Sequence
253	2p11	87,600,933	87,609,093	deletion	42	72	0.13	70	0.15	NCRNA00152
934	6p21	32,700,999	32,710,085	deletion	42	89	0.01	65	0.26	CNS^c^
1162	7q33	133,435,735	133,449,694	deletion	37	78	0.03	80	0.02	CNS
1303	8q11	51,194,577	51,195,974	deletion	21	61	0.03	50	0.12	SNTG1
1956	13q21	71,375,556	71,378,557	deletion	58	89	0.08	95	0.02	-

a CNP ID as defined in McCarroll, et al. Nature Gen 40(10):1166-74.

b *p*-value derived from chi-square analysis.

c CNS =  Conserved Noncoding Sequence, defined as a region >100bp with at least 70% similarity to sequence in mus musculus (as determined by ECR browser, ecrbrowser.dcode.org).

Four CNVs were depleted in the T1D and Twin cohorts relative to Ctrl (Table 2). The 3 CNPs are gene coding regions located on 3 different chromosomes, span 3,400 base pairs to 15,900 base pairs and are also all copy number deletions to 0 or 1. The frequency of the CNVs depleted in the diabetes cohorts range from total absence (0%) to 39%. The frequency of these CNPs in the Ctrl cohort ranged from 42%–68%. CNP1102 contains a deletion of *TYW1*, encoding a protein involved in stabilizing ribosomal decoding processes. CNP1879 is in the region coding for the ankyrin repeat and sterile alpha motif domain gene, *ANKS1B*, and the chromosome 17 CNP2240 contains coding sequence for *TRIM16*.

**Table 2:** CNVs depleted in T1D and Twin cohorts, relative to Ctrl.

CNP ID^a^	Chr	Start	End	Amplification or Deletion	Ctrl%	T1D%	Ctrl:T1D *p* b	Twin%	Ctrl:Twin *p*	Sequence
1102	7q11	66,266,764	66,282,667	deletion	68	39	0.14	10	0.001	TYW1
1879	12q23	98,319,424	98,322,865	deletion	47	22	0.20	10	0.02	ANKS1B
A588^c^	15q11	18,491,920	19,803,369	both	58	33	0.24	10	0.004	BCL8, POTEB, GOLGA6L6, GOLGA8C
2240	17p12	15,483,886	15,487,515	deletion	42	22	0.35	0	0.004	TRIM16

a CNP ID as defined in McCarroll, et al. Nature Gen 40(10):1166-74.

b *p*-value derived from chi-squared analysis.

c A588 is a novel variant identified in this study.

**Figure 2:**
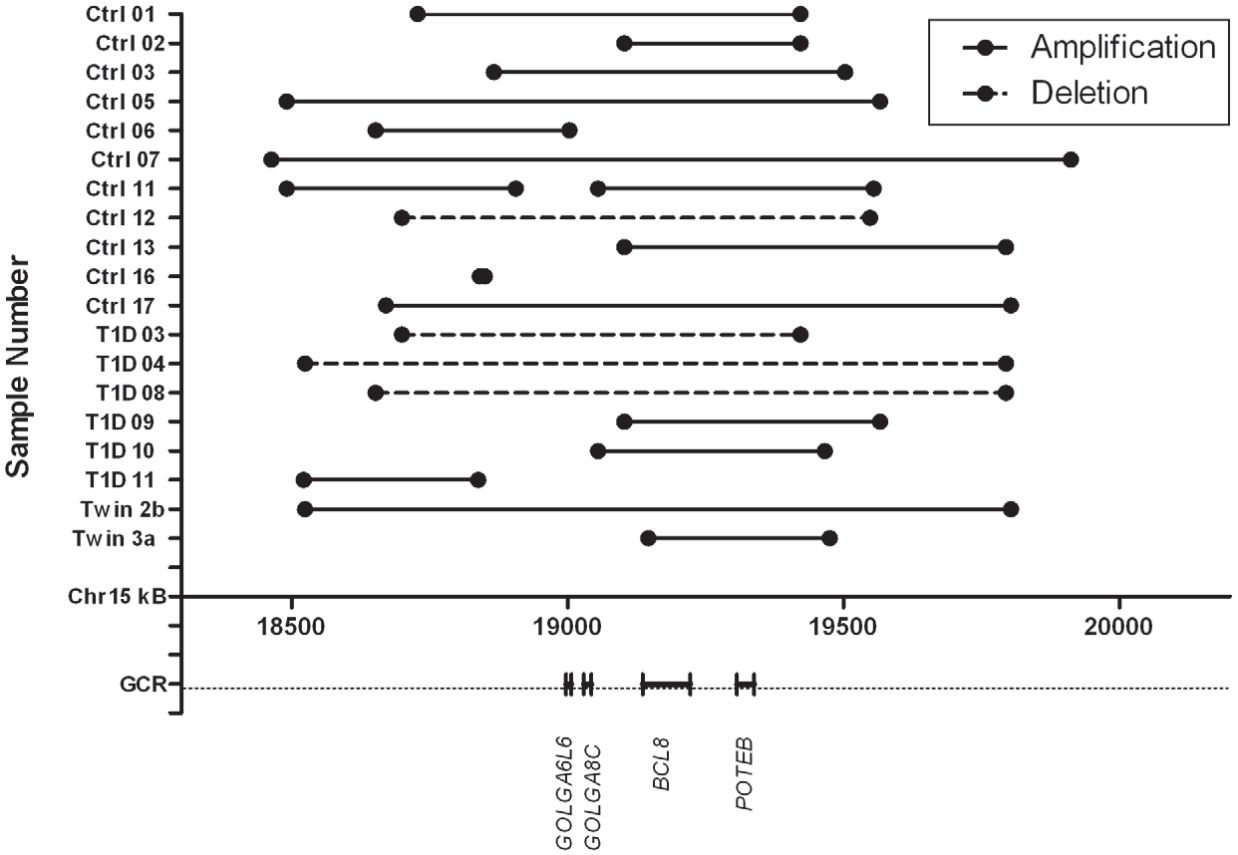
Individual breakpoints of CNVR A588. Starting and ending points of each variant in a 1.3 mega base pair region on chromosome 15. Below the x-axis are the gene coding regions (GCR) found in this portion of the genome.

The novel CNV, A588, depleted in both T1D and Twin cohorts is located on chromosome 15 and spans more than 1.3 million base pairs. It manifests as both an amplification and deletion and contains coding regions for genes like the golgin family members *GOLGA6L6* and *GOLGA8C*, B cell CLL/Lymphoma gene *BCL8* and an ankyrin domain family member, *POTEB*. The frequency of this variant in the Ctrl group is 58%, T1D group 33% and only 10% in the Twin cohort. Interestingly, many of the variants in this region do not span the entirety of the more than 1 million base pairs (Figure 2), rather we see a preponderance of overlapping and non-overlapping variants clustered in this region. Because a single variant can impact regulation and expression of a gene more than 1 megabase away, these variants were grouped together as one singular CNV region (CNVR) [19].

**Figure 3:**
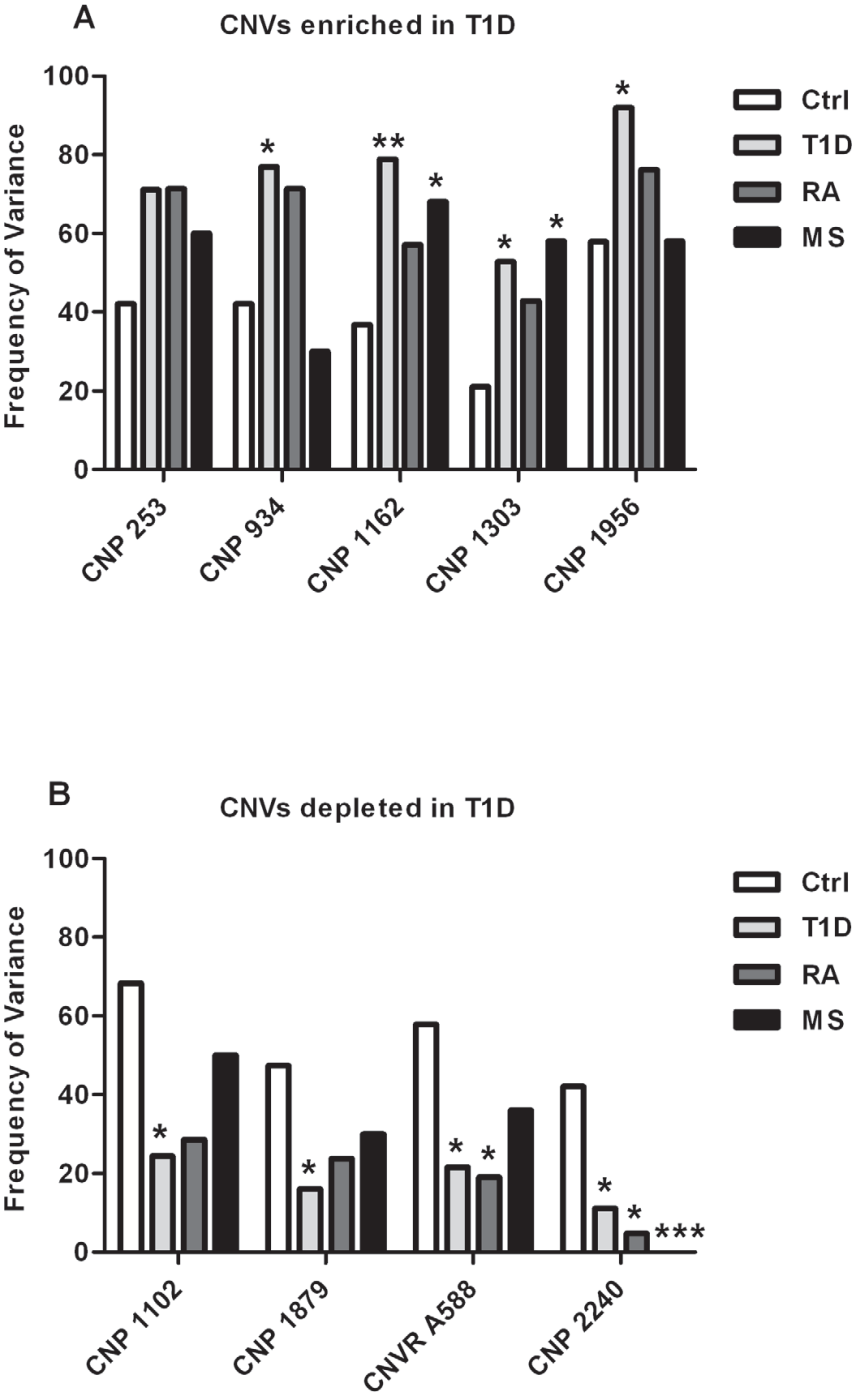
Frequencies of CNVs in other autoimmune diseases. Panel A. Frequency of CNPs identified as enriched in the T1D cohort in the Ctrl, pooled T1D, RA and MS cohorts. Panel B. Frequency of CNVs identified as depleted in the T1D cohorts, in Ctrl, pooled T1D, RA and MS cohorts. Ctrl n = 19, T1D n = 38, RA n = 21, MS n = 50. *p*-values determined by chi-square analysis, * = *p*<0.05, ** = *p*<0.005 and *** = *p*<0.0005.

The CNVs enriched and depleted in our cohorts are potentially associated with autoimmunity so we assessed the frequency of these variants in cohorts of 21 patients with rheumatoid arthritis (RA), and 50 patients with multiple sclerosis [22] (Figure 3). The T1D enriched CNPs 253, 934, 1162 and 1303 also meet the criteria for enrichment in RA; additionally, CNP1162 and CNP1303 were significantly enriched in the MS cohort (Figure 3a). The frequency of CNP1956 did not meet the criteria for enrichment in RA or MS. For those CNVs depleted in T1D, we similarly assessed their frequency in the RA and MS cohorts (Figure 3b). CNPs 1879, 2240 and the novel CNVR, A588, were also depleted in RA and MS. The depletion of CNVR A588 in RA was significant along with the depletion of CNP2240 in both RA and MS. CNP1102 was depleted in T1D and RA, but not MS (Figure 3b). Thus, a portion of CNVs enriched or depleted in T1D are found at similar frequencies in subjects with other autoimmune diseases.

**Figure 4:**
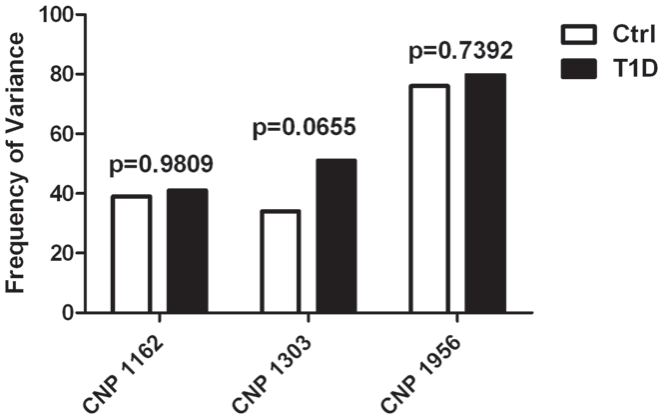
qPCR analysis of 3 T1D enriched CNPs in independent cohorts. Frequency of variance is shown for an independent Ctrl cohort (n = 73) and an independent T1D cohort (n = 73) at 3 CNP locations. Copy number was determined by ΔΔCt calculations from qPCR data. *p*-values detemined by chi-square analysis.

Finally, we sought to determine if similar differential frequency of variance could be seen in larger, independent cohorts of cases and controls. Copy number at the T1D enriched CNPs 1162, 1303 and 1956 was determined by qPCR in a group of 73 Ctrl subjects and 73 subjects with T1D, independent of previous cohorts (Figure 4). While frequency of variance did not differ appreciably between the two groups at CNPs 1162 and 1956, the difference of variance at CNP 1303 approached significance (p = 0.0655). Independent validation of 3 CNPs enriched in T1D showed one region that may continue to be of interest as a potential pathogenic variant.

## Discussion

We identified 9 CNVs enriched or depleted in 2 independent cohorts of patients with T1D or at high risk for developing disease relative to a Ctrl cohort. These CNVs represent amplifications and deletions and contain both known genes and evolutionarily conserved non-coding sequences. The regions containing these 9 CNVs were cross referenced with a list of T1D associated SNPs generated from recent reports and The National Human Genome Research Institute[9], [31], [32]. The only CNV region to have a corresponding SNP association is CNP934 located on chromosome 6p21, the major histocompatibility complex (MHC). The copy number variant region is specifically in the vicinity of HLA-DQA1; DQ alleles have long been associated with susceptibility to and protection from T1D[33]. Additionally, copy number variance in the HLA region has previously been reported in the literature[34].The duplication of this finding in a small cohort of patients indicates the importance of the MHC region on chromosome 6 in the genetic susceptibility to T1D and affirms that analysis of small sample sizes can yield biologically relevant results. Additionally, because of the limited overlap of SNPs and CNVs, this study establishes the two as independent classes of genomic variants associated with T1D.

In addition to CNP934 on chromosome 6, four other CNVs were enriched in patients with T1D and unaffected twins at high risk to develop islet-specific autoimmunity and diabetes. At least 2 of the unaffected twins already test positive for islet autoantibodies. This information indicates that the 5 CNPs enriched in these groups, while they are not beacons of clinical disease, may be involved in the formation of islet autoantibodies, or autoimmunity. Enrichment or depletion of certain of these CNPs in clinically distinct additional autoimmune diseases supports this view. CNP1303 encodes *SNTG1*, a candidate gene for scoliosis[35]. Deletions of two evolutionarily conserved non-coding sequences on chromosomes 6 and 7 could lead to dysregulation of any number of genes near the variant region on each chromosome. Underlying mechanisms by which copy number deletions at these regions and others may contribute to autoimmunity remains to be determined.

Three regions enriched in T1D were further analyzed by qPCR in independent cohorts of Ctrl and T1D patients, CNPs 1162, 1303 and 1956. CNPs 1162 and 1956 did not differ in their variance between the Ctrl and T1D independent cohorts. CNP1303 however, is variant in a greater number of T1D subjects than Ctrl and is barely shy of the criteria for enrichment with a fold change difference of 1.48. The difference in variance also approaches statistical significance. One reason the variance seen in the independent cohorts might have trended the same direction as the original data but not quite to the same degree is that our primary analysis is influenced heavily by the cohort of monozygotic twins. The affected twin of each pair was diagnosed with T1D as a child while the independent T1D cohort are adult patients with T1D who were diagnosed at varying ages. Thus, patients with an earlier onset of T1D may have a greater likelihood of possessing the CNP1303 variant.

Additionally, 4 CNVs were less likely to be variant in the T1D and Twin cohorts relative to the Ctrl group. One potential consequence of these CNVs is that normal regulation and expression of these genes contributes to the T1D disease process. Alternatively, differential expression induced by variance may confer some sort of protection to the patients in the Ctrl cohort.

CNVR A588 on chromosome 15 represents a unique class of variant. Both amplifications and deletions of varying lengths are observed with an increased frequency of variance in T1D. CNVs can affect expression levels of genes within 1 megabase of the variant through positional effects, by deletion or amplification of distal regulatory elements or other poorly understood mechanisms. An amplification of a promoter may cause increased expression of a certain gene. Deletion of an inhibitory element in that same region could also produce increased expression of that same gene. This T1D depleted CNVR contains as many as 26 gene coding regions and cDNA clones[36]. Preservation of this region at a copy number of 2 is more common in patients with T1D for unknown reasons. Determining expression levels of the genes encoded in this region could begin to unravel the mystery of this CNVR.

In considering the overlap of enrichment and depletion of these regions in additional autoimmune cohorts of RA and MS patients we can assess contributions to autoimmunity. 2 CNPs found enriched in the diabetes cohorts were also enriched in RA and may be shared regions of susceptibility to peripheral, non-neurologic autoimmunity. An additional 2 CNPs were enriched in RA and MS and could be involved in general autoimmune processes. Similarly, the 2 CNPs and 1 novel CNVR depleted in all 3 autoimmune diseases may indicate that normal functioning in these regions is also involved in general autoimmune processes.

In conclusion, 9 CNVs were found that are either enriched or depleted in 2 independent cohorts of patients with or at high risk for developing T1D. These regions may represent genetic variants contributing to islet autoimmunity or disease onset and could be used to assess risk of developing T1D. Knowledge of CNVs associated with T1D risk and islet autoimmunity could also improve our understanding of disease origins.

## Materials and Methods

### Ethics Statement

These studies were approved by the Institutional Review Board of Vanderbilt University and all subjects provided written informed consent. Monozygotic twin blood samples and family history information were provided with written informed consent using protocols and consent forms approved by the Colorado Multiple Institutional Review Board.

### Patient Recruitment

*Diabetes* is defined by the WHO criteria of classic symptoms of diabetes (polydipsia, polyuria, polyphagia and weight loss) and a plasma glucose >200 mg/dl, a fasting plasma glucose of >126 mg/dl or a 2 h plasma glucose during an oral glucose tolerance test of >200 mg/dl[1]. T1D is differentiated from type 2 diabetes by a number of criteria- history, clinical presentation and laboratory findings, including antibody testing when available. *Control* patients have never received a diagnosis of a chronic disease or syndrome and are not currently taking medication for any illness or condition. *Rheumatoid arthritis* is defined by the American College of Rheumatology Criteria. Patients displayed four or more of the following symptoms for greater than 6 months: morning stiffness, swelling in 3 or more joints, swelling of finger and/or wrist joints, symmetric swelling, rheumatoid nodules, positive rheumatoid factor, or radiographic erosions in the hand and/or wrist[37]. *Multiple Sclerosis* patients were recruited with the following characteristics: diagnosis of relapsing remitting MS (RRMS) based upon the revised McDonald criteria[38], [39], no prior cytotoxic treatments that might induce DNA damage, no family history of MS in either first or second degree relatives, and age between 25–35 (to restrict the possibility of age-related somatic mutations).

Ten pairs of monozygotic twins were selected from the Barbara Davis Center Twin Family Study, an ongoing, long-term follow up study of initially unaffected twins of patients with type 1 diabetes. Twins are ascertained through various sources, including the Barbara Davis Center Clinic, the Joslin Diabetes Clinic, the Diabetes Prevention Trial, TrialNet, and other physician and self-referrals. Family history of diabetes and other autoimmune diseases is collected at enrollment and updated over time. Serum and DNA samples are collected from twins and other family members. Serum is tested for the presence of islet autoantibodies as well as celiac and adrenal autoantibodies. Autoantibody testing is repeated for unaffected twins for as long as they remain in the study or until they develop diabetes. Twin zygosity is confirmed by testing a panel of 16 microsatellite markers. Twin DNA samples included in the present study were collected within 14 months of diagnosis of the affected twin, and at approximately the same time (within 1 week) for the two twins of each pair.

Genomic DNA samples from 73 patients with T1D, comprising the independent cohort for qPCR analysis, were obtained from Coriell Cell Repository, repository number 65895.

### Affymetrix Copy Number Variation Experiments

Peripheral blood was drawn into a Vacutainer Venous Blood Collection Tube (BD Catalog #367861) containing EDTA. Equal volume of lysis buffer (0.32M Sucrose, 10 mM Tris-HCL, 5 mM MgCl2, 0.75% Triton X-100, pH 7.6) and 2X volumes of dH20 were added to each. Samples were centrifuged and resuspended in lysis buffer. After a second centrifugation, the pellet was resuspended in proteinase K buffer (20 mM Tris-HCl, 4 mM Na2-EDTA, 100 mM NaCl, pH 7.4) and proteinase K (20 mg/ml) was added to the solution. Samples were incubated for 1 h at 55C, cooled on ice and 5.3M NaCl was added. Samples were centrifuged, supernatants kept and added to cold isopropanol and incubated for 30 minutes. Finally, genomic DNA was centrifuged and the pellet was washed twice with 70% ethanol. Genomic DNA was dissolved in Tris-HCl (pH 8.0).

Genomic DNA was hybridized to the Affymetrix Genome-Wide Human SNP Array 6.0 (Santa Clara, CA) according to the manufacturer's protocol. Following scanning, arrays were checked for quality using Affymetrix Genotyping Console. Arrays with a Contrast QC less than 0.4 were removed from further analysis. Genotypes and copy numbers were called using Birdsuite v1.5.3. As a further quality control step, arrays with an overall call rate less than 98% were discarded from further analysis.

### Copy Number Analysis

Genomic regions with a Birdsuite copy number call confidence value less than 5 were merged to the adjacent region with a confidence score greater than 5, assuming the copy number of that confident region. Next, each genome was narrowed down to a list of genomic variants with confidence scores greater than 5. Regions of CN = 2 were discarded. These lists were merged with the list of CNPs and regions of variance not represented by a CNP were denoted as “novel” [28]. Next, CNVs that were present in greater than 40% of the T1D group at a 1.5 fold change greater frequency as compared to the Ctrl group were determined to be enriched and CNVs present in greater than 40% of the Ctrl group at a 1.5 fold change greater frequency as compared to the T1D group were determined to be depleted. For validation of these CNVs whose presence or absence may be associated with diabetes, an identical analysis was performed on the discordant twin cohort, as compared to Ctrl and with the additional step of comparing the affected twins versus their unaffected co-twin pair to determine any variants present differentially within the group. CNVs enriched or depleted in both the twins and unrelated T1D adults were selected for further analysis. Chi-square analyses were performed on each CNV of interest in each cohort versus control based on the presence or absence of variance.

We assessed statistical significance for the observed overlapping CNVs in both T1D cohorts relative to the Ctrl group using a permutation test with 1000 permutations. Briefly, keeping the number of patients fixed in each of the three groups, we randomly permutated group status for the samples (so that they were re-assigned to different groups) and re-calculated the number of enriched CNVs in both disease groups relative to Ctrl. This process was repeated 1000 times. The p-value for the number of observed overlapping CNVs (i.e. 10) was estimated by the number of permutations with 10 or more overlapping CNVs divided by the total number of permutations.

### Quantitative PCR Experiments

To validate the copy number of variant regions from the Affymetrix chip, primer assays were ordered from Applied Biosystems, selected from their inventoried stock of assays designed specifically to detect genomic copy number. Reactions were run with 20 ng genomic DNA per the standard Applied Biosystems protocol in a 7300 Real Time PCR System. All samples were run in triplicate with a multiplexed RNase P reference and copy number was called using Applied Biosystem's CopyCaller v.1.0.

## Supporting Information

Table S1 **Raw Copy Number Calls derived from Affymetrix SNP 6.0 Arrays.** Copy numbers were called across the genome using the Birdsuite program. Tabs separate calls by group: Control contains calls for the 19 unrelated adult control subjects, Type 1 Diabetes contains calls for the 18 unrelated adults with T1D, and Twin contains calls for the 10 pairs of monozygotic twins, each pair denoted by the same number with letter a or b. Additionally, confidence values below 5 are assumed to be invalid calls in these studies. (XLS)

Table S2 **Comparisons of Birdsuite Copy Number Calls to qPCR calls.** 185 unique gene region-sample comparisons were made between the copy number as determined by Birdsuite based on Affymetrix array data and copy number determined independent of the array by qPCR. (XLS)
